# Reliability and validity of two fitness tracker devices in the laboratory and home environment for older community-dwelling people

**DOI:** 10.1186/s12877-018-0793-4

**Published:** 2018-05-03

**Authors:** Elissa Burton, Keith D. Hill, Nicola T. Lautenschlager, Cecilie Thøgersen-Ntoumani, Gill Lewin, Eileen Boyle, Erin Howie

**Affiliations:** 1 School of Physiotherapy & Exercise Science, Curtin University, Perth, Australia; 2 Department of Psychiatry, Academic Unit for Psychiatry of Old Age, The University of Melbourne and NorthWestern Mental Health, Melbourne Health, Melbourne, Australia; 3 School of Psychology, Curtin University, Perth, Australia; 4 School of Nursing, Midwifery & Paramedicine, Curtin University, Perth, Australia; 5 Department of Health, Human Performance & Recreation, University of Arkansas, Fayetteville, USA

**Keywords:** Ageing, Activity tracker, Accelerometer

## Abstract

**Background:**

Two-thirds of older Australians are sedentary. Fitness trackers have been popular with younger people and may encourage older adults to become more active. Older adults may have different gait patterns and as such it is important to establish whether fitness trackers are valid and reliable for this population.

The aim of the study was to test the reliability and validity of two fitness trackers (Fitbit Flex and ChargeHR) by step count when worn by older adults. Reliability and validity were tested in two conditions: 1) in the laboratory using a two-minute-walk-test (2MWT) and 2) in a free-living environment.

**Methods:**

Two 2MWTs were completed while wearing the fitness trackers. Participants were videoed during each test. Participants were then given one fitness tracker and a GENEactiv accelerometer to wear at home for 14-days.

**Results:**

Thirty-one participants completed two 2MWTs and 30 completed the free-living procedure. Intra Class Correlation’s of the fitness trackers with direct observation of steps (criterion validity) was high (ICC:0.86,95%CI:0.76,0.93). However, both fitness trackers underestimated steps. Excellent test-retest reliability (ICC ≥ 0.75) was found between the two 2MWTs for each device, particularly the ChargeHR devices. Good strength of agreement was found for total distance and steps (fitness tracker) and moderate-to-vigorous physical activity (GENEactiv) for the free-living environment (Spearman Rho’s 0.78 and 0.74 respectively).

**Conclusion:**

Reliability and validity of the Flex and ChargeHR when worn by older adults is good, however both devices underestimated step count within the laboratory environment. These fitness trackers appear suitable for consumer use and promoting physical activity for older adults.

## Background

Almost half of middle aged (45–64 years) Australians are physically inactive and this increases to 66% for older Australians (aged 65+ years) [1]. Between 2004 and 2012 there was a 20% increase in sedentary behaviour for older adults living in Australia [1]. Insufficient levels of physical activity lead to many health issues such as high blood pressure, increased risk of a myocardial infarction, some types of cancer and type 2 diabetes [2, 3]. More specifically for older people, a reduction in physical activity can lead to a decrease in physical function [4] and an increase in falls [5, 6]. This can limit how often the older person leaves their home to see family and friends, go shopping for food, and can ultimately affect their ability to maintain daily activities, which are essential to living independently.

Due to the decline in physical activity as people age, researchers are investigating different ways to motivate older people to become more active and better understand their activity levels. Accelerometers or pedometers have predominantly been used in the past to measure levels of activity such as work, physical activity and socialising across a period of time (e.g. 7 days) [7–9]. Both these instruments have limitations for the end user: pedometers are usually worn on a belt around the waist and only count steps and accelerometers are expensive and primarily used by researchers to measure physical activity intensity, such as in METs (Metabolic Equivalent of Tasks) and patterns of activity throughout the day. However, during the last decade a number of new commercial devices have been introduced onto the market called fitness trackers (or activity monitors). Over 102 million fitness trackers and smart watches (which include fitness measures) were sold globally in 2016 alone [10]. There are a number of companies manufacturing fitness trackers, and the majority link their device to a free app which provides more detailed information for the user (such as step count, activity minutes, stairs, sleep, heart rate), as well as the direct feedback on the device. The purpose of fitness trackers is to encourage people to be more active by monitoring their progress throughout the day. The popularity of these commercial fitness trackers has grown at a rapid rate and there is a growing body of research to determine the reliability and validity of these devices for different populations.

However, while there has been growth in testing the validity and reliability of fitness trackers with older people over the past few years [11] the evidence is still limited, particularly compared to other younger populations [11]. Studies are needed that investigate older people’s use of these devices not only in the laboratory, where gait patterns may be changed for a short period of time, but also in their home environment where older adults may revert to usual habits (i.e. more ecologically valid). It is known that many older people reduce their activity levels compared to younger healthier populations. They may also use a walking aid and are less likely to lift their feet when turning, all of which may affect the data recordings of the fitness trackers. If fitness trackers are to be used in studies to establish whether they can promote an increase in physical activity in older people, it is necessary to know whether these devices are reliable and valid in this population. It will also be of benefit to older adults to know whether these commercially available devices are able to provide accurate activity data.

The aim of this study was to test the reliability and validity of two fitness trackers (Fitbit Flex and Fitbit ChargeHR: Fitbit, Inc.; San Francisco, United States of America) by step count when worn by older community-dwelling people. Reliability and validity were tested under the following conditions, which occurred on separate occasions:

1. Two 2 Minute Walk Tests (2MWTs) in the laboratory, and
2. 14-day free-living period where usual daily activities were completed.

## Methods

### Design

Step data were collected in both controlled (laboratory) and free-living (home) environments. In the laboratory environment, the number of steps recorded on the trackers was compared with the number of steps counted by two observers (via video), which is considered the ‘gold standard’; while in the free-living environment a range of activity data recorded on the fitness tracker devices was compared with data from a research-grade, wrist-worn accelerometer over a 14-day period. Ethics approval was obtained from the University Human Research Ethics Committee (RDHS-197-15).

### Participants

Participants were older community-dwelling adults living in the Perth (Western Australia) metropolitan area. They were recruited through convenience sampling through: advertisements on a local radio station and in a seniors’ electronic newsletter; posters; and, by word-of-mouth in a local retirement village. The inclusion criteria were: i) aged 65 years and over; (ii) living in Perth; (iii) owns a smart phone or tablet; (iv) understands English and (v) no medical condition which made participation in the study unsafe (i.e. must be able to walk for a minimum of 2 minutes unassisted). The same participants took part in both the laboratory and free-living tests.

### Recruitment process

Participants contacted the researchers directly by phone after learning about the study from one of the various forms of advertising described above. Each participant received an information sheet and consent form (either via mail or email) and were given the opportunity to ask questions prior to providing written, informed consent. They were scheduled to meet at the University laboratory where their demographic data and an additional self-report tool for determining physical activity for the past week was collected using the Physical Activity Scale for the Elderly (PASE) [12]. The PASE is a 12-item questionnaire designed to assess physical activity levels of older people over a seven-day period [12]. Physical activity included leisure, household and occupational activity, with an overall PASE score calculated for each participant [12]. PASE scores usually range between 0 and 400, with higher scores indicating higher levels of physical activity [12]. The PASE is a reliable and valid tool for use face-to-face with older adults and was used as a secondary outcome for the 14-day data collection [13]. After completing the PASE, participants then undertook the two 2MWTs.

### Fitness trackers

Two types of commercial (Fitbit) fitness trackers were used during the laboratory testing (2 x 2MWTs): the Flex (released May 2013) and the ChargeHR (released October 2014). These Fitbit devices use a tri-axial accelerometer to measure movement, the raw data modes of the Fitbit devices are not available to end users and the devices provide real time data. The Flex gives information to the user by an additional light lighting up for every 2000 steps undertaken over a day, to a total of 5 lights (i.e. 10,000 steps); more specific activity data are available on the Fitbit app. The ChargeHR gives an actual step count on the device as well as heart rate, physical activity minutes and stairs. Further data can also be accessed on the app such as for sleep. Step count was the primary outcome used to assess reliability and validity of the devices during the two 2MWTs. After completing the laboratory trials, participants were randomly allocated either the Flex or ChargeHR for 14-days. Allocation of the fitness tracker type for the 14-day period was randomised by a researcher not involved in data collection, using an electronic random number generator. It was not possible for participants or those collecting data to be blind to the type of tracker because the trackers are substantially different in appearance and the type of fitness tracker is named on the Fitbit app. Step count, physical activity minutes, total distance and sleep were all used as outcome measures during the 14-day free-living period.

### Direct observation

For the laboratory trial each 2MWT for every participant was video recorded. The mean of the number of steps counted by two independent observers of the video recording of the 2MWT was then used as the ‘gold standard’ for actual steps taken during the test [14]. Fulk et al. (2014) had their researchers count the number of steps taken from a video on two separate occasions separated by at least a week (test-retest reliability) and found the Intra Class Correlation (ICC) agreement between the two counts to be 0.99 [14]. The same procedure was followed in this present study for both 2MWT one (2MWT(1) and 2MWT two (2MWT(2)) with 7 days between counts. Test-retest reliability between the two 2MWTs, as calculated using an ICC, was found to be 0.99 (0.99–1.00) with Rater 1 counting an average of 225 steps (SD: 18.8) on day 1 and 7 for 2MWT(1) and Rater 2 counting an average 224 steps (SD: 18.7) respectively. 2MWT(2) had a test-retest ICC of 1.00 (0.99–1.00) with Rater 1 and Rater 2 both counting an average of 228 steps (SD: 18.5). These analyses therefore demonstrate the reliability of the ‘gold standard’.

### Accelerometers

GENEactiv (wrist worn) accelerometers were also worn during the 2MWT and over the 14-day period on the same wrist as the fitness tracker. The GENEactive is a triaxial, + 6 g seismic acceleration sensor, has 500 MB of memory and can store 8d of data in raw mode with 12-bit resolution [15]. The GENEactiv records movement at 30hz and was collapsed into 60 s epochs for data processing. The previously validated GENEactiv [15] was used as the ‘gold standard’ measure for the 14-day free-living part of the study. The GENEactiv has been shown to be a valid and reliable measurement tool firstly using a multi-axis shaking table to determine technical reliability and validity and secondly criterion validity against relative VO_2_ [15]. It was reported as being capable of classifying different intensities of physical activity in adults and therefore physical activity minutes [15]. Moderate-to-Vigorous Physical Activity (MVPA), total physical activity and sleep in minutes per day were all measured using the accelerometer. It must be acknowledged that the GENEactiv may not be ‘gold standard’ for measuring sleep. However, this is not the aim of the study.

### Procedures

#### 2 Minute walk test

The 2MWT was completed twice by participants. The 2MWT is a valid and reliable test and has been used with a number of older populations [16, 17]. The 2MWT requires the participant to stand behind a line (tape on the floor) and then walk without assistance as fast and safe as permissible for 2 minutes in a hallway or somewhere outside, as long as the walking space/path is clear of obstacles and well lit. For this study, participants were asked to sit on a chair situated behind the starting line of an 8 metre walking path that was measured prior to each data collection session. Each Fitbit device worn by a single participant had a different coloured band and was individually numbered to avoid confusion. The two researchers collecting the data attached two Flex (one blue, one black) and one GENEactiv accelerometer to the left wrist and two Fibit ChargeHR (one purple, one black) and one GENEactiv accelerometer to the right wrist of each participant. The GENEactiv accelerometers were also worn for the participants to familiarise themselves with the device but data were not collected from this device in this instance.

Participants were asked to stand (immediately behind the starting line) and be still while the number of steps for each of the four Fitbit devices was recorded and the start time noted. Following demonstration by the researcher each participant was asked to walk as far as possible along the designated 8 metre track (turning carefully at the 8 metre mark shown by tape on the floor) for 2 minutes, following standard 2MWT participant instructions. If participants used a walking aid prior to participating in the study they were asked to use it during testing. The second researcher video recorded the steps taken by the participant. On completion of 2 minutes the participant was asked to stand still while each Fitbit device was synced to a tablet Fitbit app and the step count recorded. To calculate the number of steps taken to complete the 2MWT(1), the recording of number of steps taken on the Fitbit device prior to starting the test for each device was subtracted from the recording of number of steps taken at test completion. After a 5 minute rest 2MWT(2) was conducted under the same conditions. Each fitness tracker step count was used to determine validity and reliability during the 2MWT.

#### 14-day free-living measurements

After completing the two 2MWTs participants were provided with a randomly allocated Flex or ChargeHR fitness tracker and an accelerometer to wear for 14-days (including sleeping). The only exception was to remove when in water (e.g. shower or swimming). Each participant was given instructions (written and verbal) on how to use the allocated fitness tracker and app (it was downloaded for them where possible) and included instructions on how to charge it (every second day). Each participant was given a choice of which wrist they preferred to wear the fitness tracker and accelerometer on and were asked to keep it on that same wrist for 14-days and to complete their daily routines as usual. After 14-days a researcher collected the fitness tracker, accelerometer and repeated the PASE with each participant.

### Analysis

IBM SPSS for windows, version 24 (Armonk, NY) and/or Stata/IC 14.1 (StataCorp LP, College Station TX, USA) were used to analyse the data and a *p*-value of <.05 was considered statistically significant.

### Demographics

The demographic data including health status, prescribed medications, living arrangements, education, physical activity (using PASE) and mobility status were summarised using descriptive statistics.

### Reliability – 2MWT

Inter-rater reliability of the fitness tracker devices was analysed using the ICC for each of the 2MWTs. Test-retest reliability between 2MWT(1) and 2MWT(2) for each device was assessed by calculating ICC (two-way absolute agreement, single measures, 95% confidence interval) [18]. Paired sample t-tests were used to examine the differences in mean step count for each device [19].

### Criterion validity – 2MWT

The ICC (two-way, absolute agreement, single measures, 95% confidence interval) was used to examine criterion validity between the fitness tracker devices (step count) and the actual steps taken during each 2MWT determined by the video count (‘gold standard’). ICC ratings were ≥ 0.75:excellent, 0.60–0.74: good, 0.40–0.59: fair and < 0.40: poor [20]. The Bland-Altman method was used to examine the limits of agreement between the devices’ estimated steps and actual steps taken, that is: the differences between the fitness tracker estimated steps and actual steps taken were plotted against the average measures obtained by the two methods (fitness trackers and video observations) [14, 21–23]. The mean difference and 95% confidence intervals are illustrated using horizontal lines, which assist in identifying systematic trends or outliers [14, 24].

### Validity – 14-day free-living

As activity data deviates from normality, Spearman correlations were used to compare daily physical activity minutes, total distance travelled, step count and sleep for the fitness tracker (Fitbit) devices and accelerometer (GENEactiv) over the 14-days. It was hypothesised that there would be a strong (> 0.7) positive correlation, indicating validity, between the measures for step count and physical activity. As physical activity is an episodic behaviour and averaging activity metrics over 14-days would eliminate the actual variation within each individual, correlations between daily Fitbit metrics and GENEactiv metrics were calculated within each individual and then analysed across participants. The mean of those correlations (and range) were then calculated and the proportion of the correlations that were significant at the 0.05 level for each pairwise correlation are presented in the results. To determine if demographics and other behavioural variables were associated with the strength of correlation between the fitness tracker steps and GENEactiv MVPA and sleep for the 14-day free-living data were examined using t-tests and ANOVAs for categorical predictors (e.g. type of Fitbit, arm worn, gender) and Spearman correlations for continuous variables (e.g. age, body mass index, distance walked in 2MWT). Strength of correlations for these data can be interpreted as: 0.80–1.0 very strong, 0.60–0.79 strong, 0.40–0.59 moderate, 0.20–0.39 weak and 0.00–0.19 very weak. [25]

## Results

Thirty one participants, with an average age of 74.2 years (SD:5.8) completed the demographic data collection and laboratory tests (2 x 2MWTs). One participant withdrew after 12 h of the free-living data collection, due to feeling stressed about wearing the devices. Data from this participant were not included in the 14-day analysis. Participant characteristics are shown in Table 1, and two-thirds of the participants were female. Three-quarters had at least one health issue and 80% were taking at least one medication prescribed by a Doctor. The majority of participants had no trouble walking (defined by self-report). The average PASE score for the group was 129 (SD:83.3). All participants were able to understand the instructions on how to use the fitness trackers and did not display signs of Dementia or cognitive impairment. However, they were not formally assessed for cognitive status.

**Table 1:** Participant characteristics

Variable	Participants (*n* = 31)
Age (years) M(SD)	74.2 (5.78)
Sex (male n (%): female n (%))	11 (35.5): 20 (64.5)
Height (cm) M(SD)	168.9 (8.6)
Weight (kg) M(SD)	75.2 (14.8)
Marital status n (%)	
Never married	4 (12.9)
Married/De facto	18 (58.1)
Widowed	3 (9.7)
Separated/Divorced	6 (19.4)
Living arrangements n (%)	
Alone	11 (35.5)
With spouse/partner	20 (64.5)
Education n (%)	
High school	14 (45.2)
Trade	5 (16.1)
Tertiary	12 (38.7)
Health issues n (%)	23 (74.2)
Prescribed medications n (%)	24 (80.0)
Mobility n (%)	
No trouble walking	28 (90.3)
Some trouble but does not use walking aid	2 (6.5)
Needs a walking stick outside	1 (3.2)
Falls in last year n (%)	7 (24.1)
PASE M(SD)	129.9 (83.3)

Note. M Mean, *SD* standard deviation, *PASE* Physical Activity Scale for the Elderly, *cm* centre metres, *kg* kilogram

### Laboratory data: 2 Minute walk tests

Table 2 presents the ICCs and 95% Confidence Intervals (95%CI) for the steps measured by the four fitness tracker devices compared to observer step count. More steps (225) were counted by direct observation compared to the four fitness trackers (ranged from 196 steps: Flex black to 219 steps: ChargeHR black). The ICC comparing direct observation with the Flex’s for the 2MWT(1) and 2MWT(2) were excellent at 0.77 (95%CI: 0.57–0.88) and 0.76 (95%CI: 0.53–0.88) respectively. ICC’s comparing the ChargeHR’s to direct observation were higher than those of the Flex, but still within the excellent rage; 2MWT(1) was 0.95 (95%CI: 0.92–0.97) and 2MWT(2) 0.90 (95%CI: 0.83–0.95). Overall all of the fitness tracker devices counted fewer steps than were actually taken by the participants compared to the ‘gold standard’ video observation counts.

**Table 2:** *Comparing fitness tracker devices to direct observation for steps*

Fitness Tracker (M(SD) steps)	Direct observation^a^	Flex blue	Flex black	Flex ICC (95%CI)	ChargeHR purple	ChargeHR black	ChargeHR ICC (95% CI)
2MWT(1)	225 (18.8)	198 (30.9)	196 (23.9)	0.77 (0.57,0.88)	217 (28.7)	219 (25.3)	0.95 (0.92,0.97)
2MWT(2)	228 (18.5)	195 (28.8)	198 (26.5)	0.76 (0.53,0.88)	216 (29.0)	219 (29.5)	0.90 (0.83,0.95)

Note. M mean, *SD* standard deviation and ICC: Intra Class Correlation, 95%CI is 95% Confidence Interval

^a^From Rater 1

Table 3 presents fitness tracker comparisons between the two 2MWT. Excellent test-retest reliability (ICC ≥ 0.75) for each fitness tracker type was found between the two 2MWT. The greatest variability for the 95%CI was shown for the Flex devices. The two ChargeHR devices had the highest reliability across the two tests.

**Table 3:** *Test-retest reliability, comparing fitness tracker devices between 2MWT(1) and 2MWT(2)*

2MWT(1) versus 2MWT(2)	Mean Difference (95%CI)	ICC (95%CI)
Number of steps (Flex blue)	2.87 (−6.26, 12.00)	0.79 (0.57, 0.90)
Number of steps (Flex black)	−2.13 (−8.46, 4.20)	0.87 (0.73, 0.94)
Number of steps (ChargeHR purple)	1.32 (−2.97, 5.61)	0.96 (0.91, 0.98)
Number of steps (ChargeHR black)	0.00 (−5.50, 5.50)	0.92 (0.84, 0.96)
Distance walked	−3.26 (−4.44, −2.07)	0.98 (0.85, 0.99)

*Note*. 2MWT(1): 2 Minute Walk Test number 1, 2MWT(2): 2 Minute Walk Test number 2, ICC: Intra Class Correlation, 95%CI: 95% Confidence Interval. Distance walked was measured by laps undertaken (8 m distance) plus distance from tape for final lap (m)

Table 4 presents the inter-device reliability for the fitness tracker devices for the 2MWT(1). In general there was an excellent level of agreement between the same devices i.e. Flex versus Flex, however the ICC reduced markedly when comparing different types of devices i.e. Flex versus ChargeHR. Similar results were found for both 2MWT, therefore the 2MWT(2) results are not presented.

**Table 4:** Inter-device reliability, comparison of steps between the fitness trackers devices during 2MWT(1)

2MWT(1)	Mean Difference (95%CI)	ICC (95%CI)
Flex blue vs Flex black	2.5 (− 4.2, 9.2)	0.88 (0.75, 0.94)
ChargeHR purple vs ChargeHR black	−1.65 (− 5.6, 2.3)	0.96 (0.92, 0.98)
Flex blue vs ChargeHR purple	−18.65 (− 29.27, −8.02)	0.62 (0.15, 0.82)
Flex blue vs ChargeHR black	− 20.23 (− 30.83, −9.75)	0.56 (0.04, 0.79)
Flex black vs ChargeHR purple	−21.16 (− 30.74, − 11.59)	0.56 (− 0.01, 0.80)
Flex black vs ChargeHR black	−22.81 (−32.41, − 13.21)	0.47 (− 0.11, 0.75)

*Note*. 2MWT(1): 2 Minute Walk test number 1, ICC: Intra Class Correlation, 95%CI: 95% Confidence Interval

**Fig. 1:**
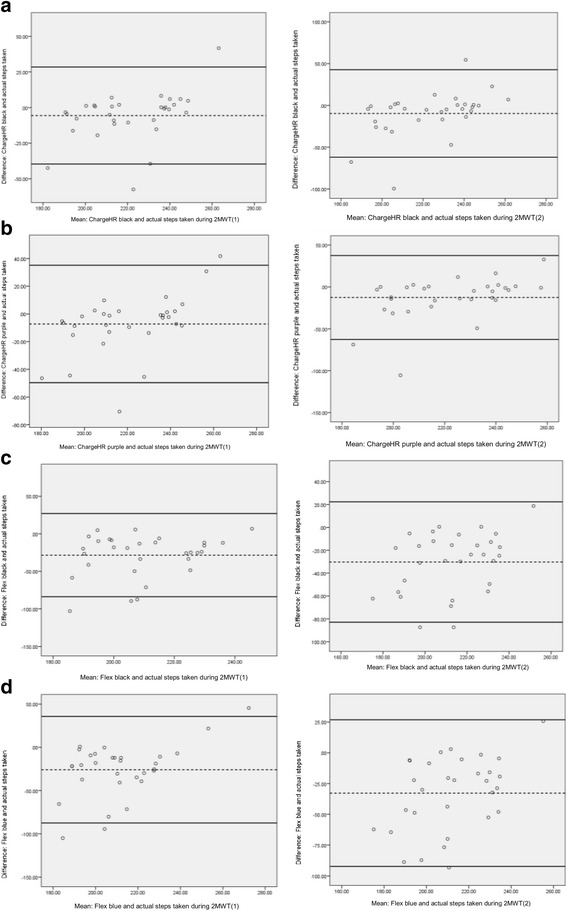
Bland-Atman plots: **a** Bland-Altman plot demonstrating step count agreement between actual steps taken counted from the video and ChargeHR black estimated steps for 2MWT(1) and 2MWT(2). **b** Bland-Altman plot demonstrating step count agreement between actual steps taken counted from the video and ChargeHR purple estimated steps for 2MWT(1) and 2MWT(2). **c** Bland-Altman plot demonstrating step count agreement between actual steps taken counted from the video and Flex black estimated steps for 2MWT(1) and 2MWT(2). **d** Bland-Altman plot demonstrating step count agreement between actual steps taken counted from the video and Flex blue estimated steps for 2MWT(1) and 2MWT(2). Dashed line is the mean difference and the solid bold lines the 95% confidence intervals for each diagram. Note that the scale on the y-axis is not the same between the graphs

**Fig. 2:**
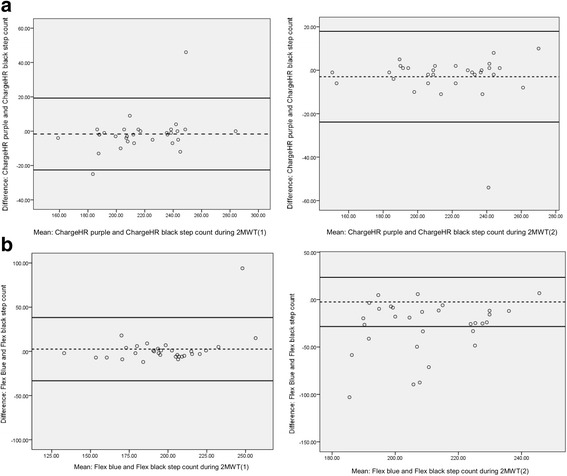
Bland-Atman plots: **a** Bland-Altman plot demonstrating step count agreement between estimated steps taken counted from the ChargeHR purple and ChargeHR black estimated steps for 2MWT(1) and 2MWT(2). **b** Bland-Altman plot demonstrating step count agreement between estimated steps taken counted from the Flex blue and Flex black estimated steps for 2MWT(1) and 2MWT(2). Dashed line is the mean difference and the solid bold lines the 95% confidence intervals for each diagram. Note that the scale on the y-axis is not the same between the graphs

The distribution of error and testing for proportional bias is shown in the Bland-Altman plot analyses [26]. The plots presented in Figs. 1 and 2 show the residuals of the various step count estimates on the y-axis in relation to the mean of the two methods on the x-axis. In Fig. 1 the narrowest 95% limits of agreement were for both ChargeHR devices; ChargeHR Black/observer count 2MWT(1) (difference = 68.2); ChargeHR Purple/observer count 2MWT(1) (difference = 84.8); ChargeHR Purple/observer count 2MWT(2) (difference = 100.0); ChargeHR Black/observer count 2MWT(2) (difference = 104.8). For the Flex devices 95% limits of agreement were from Flex black 2MWT(1) (difference = 110.8), 2MWT(2) (difference = 119.2); to Flex blue 2MWT(1) (difference = 123.0) and 2MWT(2) (difference = 123.0).

Figure 2 compares the fitness tracker devices. The narrowest 95% limits of agreement were for the ChargeHR devices 2MWT(1) (difference = 41.9), 2MWT(2) (difference = 41.8) compared to the Flex devices 2MWT(1) (difference = 71.6), and 2MWT(2) (difference = 51.8).

Significant proportional bias was found for ChargeHR Black/observer count 2MWT(1) (slope = 0.166, *p* = 0.023) and 2MWT(2) (slope = 0.231, *p* = 0.006), Charge Purple/observer count 2MWT(1) (slope = 0.255, *p* = 0.004) and 2MWT(2) (slope = 0.255, *p* = 0.007), Flex Black/observer count 2MWT(2) (slope = 0.133, *p* = 0.043), Flex Blue/observer count 2MWT(1) (slope = 0.228, p = 0.007) and 2MWT(2) (slope = 0.183, *p* = 0.017) and Flex Black/Blue 2MWT(1) (slope = 0.162, *p* = 0.025). There were non-significant proportional bias for Flex Black/observer count 2MWT(1) (slope = 0.059, *p* = 0.189), ChargeHR Purple/Black 2MWT(1) (slope = 0.105, *p* = 0.075) and 2MWT(2) (slope = 0.002, *p* = 0.795) and Flex Black/Blue 2MWT(2) (slope = 0.032, *p* = 0.338).

### Free-living environment data: 14-days

Correlations between the variables between the devices for 14-days of free-living activity are presented in Table 5. Strength of agreement ranged from very weak (physical activity minutes and total physical activity), weak for sleep, through to strong for Fitbit total distance and GENEactiv MVPA and steps, respectively (most of the individual correlations for these analyses were significant – see Table 5). It must be noted that 16 of the 30 fitness tracker devices during the 14-day data collection phase had some missing data (physical activity minutes and sleep) and/or data that underestimated physical activity minutes when compared to step count that was implausible. Missing data were eliminated in the analysis but data that may have underestimated were not. When excluding these data (PA minutes and sleep) all other variables had moderate to good validity compared to the accelerometer, when worn in a free-living environment.

**Table 5:** Correlations between the fitness trackers and accelerometer for the 14-day free living data

Fitness tracker & accelerometer variables	n	Spearman Rho (range)^a^	% of correlations ≤ 0.05
Physical activity minutes & Moderate-to-vigorous physical activity	28 (14 Flex, 14 ChargeHR)	0.48 (− 0.49, 0.89)	46.4
Steps & Moderate-to-vigorous physical activity	29 (14 Flex, 15 ChargeHR)	0.70 (− 0.10, 0.96)	75.9
Total distance & Moderate-to-vigorous physical activity	29 (14 Flex, 15 ChargeHR)	0.72 (0.01, 0.96)	79.3
Physical activity minutes & total Physical activity	28 (14 Flex, 14 ChargeHR)	0.14 (−0.31, 0.60)	3.6
Steps & total Physical activity	29 (14 Flex, 15 ChargeHR)	0.54 (−0.12, 0.90)	58.6
Total distance & total Physical activity	29 (14 Flex, 15 ChargeHR)	0.53 (−0.13, 0.90)	58.6
Sleep (fitness tracker) & Sleep (Geneactiv accelerometer)	24 (10 Flex, 14 ChargeHR)	0.37 (−0.21, 1.00)	25.0

*Note*. Fitness tracker device variable named first, accelerometer variable second. ^a^Mean and range of up to 29 participant correlations

Table 6 presents the individual correlations between steps (fitness tracker) and MVPA (accelerometer). Those who fell in the past year had a significantly lower correlation between the fitness tracker and accelerometer activity. No other variables were significant, including the type of fitness tracker device worn or which arm they were worn on (dominant or non-dominant).

**Table 6:** Factors associated with fitness tracker steps and GENEactiv moderate-to-vigorous physical activity and sleep 14-day free-living data

		Correlation between Fitness Tracker Steps & GENEactiv Moderate-to-vigorous physical activity		Correlation between fitness tracker and GENEactiv Sleep	
		Mean Correlation (SD)	*p*-value	Mean Correlation (SD)	*p*-value
Categorical Variables					
Fitness tracker	*Flex*	0.68 (0.27)	0.674	0.47 (0.27)	0.220
	*ChargeHR*	0.72 (0.29)		0.30 (0.35)	
Arm worn	*Dominant*	0.66 (0.32)	0.434	0.38 (0.35)	0.817
	*Non-Dominant*	0.75 (0.22)		0.35 (0.31)	
Gender	*Male*	0.73 (0.19)	0.683	0.23 (0.33)	0.133
	*Female*	0.68 (0.33)		0.44 (0.31)	
Age group	*65–69*	0.86 (0.12)	0.313	0.35 (0.36)	0.705
	*70–79*	0.66 (0.32)		0.41 (0.32)	
	*80–89*	0.66 (0.24)		0.25 (0.36)	
Living status	*Alone*	0.62 (0.35)	0.332	0.44 (0.18)	0.446
	*With Spouse*	0.74 (0.25)		0.33 (0.39)	
Health Issues	*No*	0.76 (0.18)	0.540	0.31 (0.37)	0.634
	*Yes*	0.68 (0.30)		0.39 (0.32)	
Prescribed Medications	*No*	0.75 (0.20)	0.703	0.41 (0.40)	0.728
	*Yes*	0.69 (0.30)		0.35 (0.32)	
Fall in past year	*No*	0.75 (0.20)	0.040*	0.38 (0.35)	0.716
	*Yes*	0.49 (0.45)		0.32 (0.19)	
Problems staying	*No*	0.70 (0.23)	0.937	0.48 (0.33)	0.081
asleep	*Yes*	0.70 (0.35)		0.24 (0..29)	
Continuous Variables					
		Correlation	p-value	Correlation	p-value
Age		−0.32	0.086	−0.004	0.984
Body Mass Index		0.15	0.279	−0.05	0.823
Distance walked in 2MWT(1)		0.12	0.520	−0.15	0.482
Distance walked in 2MWT(2)		0.17	0.368	−0.18	0.400
PASE (baseline)		0.29	0.148	−0.167	0.459
Post_PASE		.030	0.876	0.077	0.721

Note. 2MWT: two minute walk test, PASE: Physical Activity Scale for the Elderly. All 30 fitness tracker device data were utilised in the step data however only 10 Flex and 14 ChargeHR devices were utilised in the sleep analysis data. *p*-values for categorical variables from ANOVA or t-test comparing correlations between groups. *statistically significant at *p*-value <0.05

## Discussion

As people age there is a tendency to decrease the amount of physical activity undertaken. Commercial fitness trackers were created to assist people in monitoring their daily physical activity and sleep with a view to potentially help motivate them to be more active. This study investigated the reliability and validity of two fitness trackers (Flex and ChargeHR), when worn by older adults in both the laboratory and free-living environment and found both to be reliable and valid in measuring physical activity in older adults. Many studies have only looked at either laboratory based [27–29] or free-living [30–32]. Only one study to date has combined both environments using older adults as participants and they did not use either the Flex or ChargeHR (they used One or Zip) [33].

The Flex devices measured fewer steps than direct observation, ranging from 27 to 33 steps difference in 2MWT(1) and 2MWT(2). This needs to be considered in future studies that wish to utilise the Flex for identifying distances travelled or promoting activity because it appears to underestimate total steps taken. However, the ICC’s when comparing the fitness trackers to direct observation were high. Other studies validating the step count of the Flex with direct observation in younger adults found similar results to the present study, with the Flex underestimating the step count, with high ICC agreement found [18, 19, 27]. A validation study with older people asked to walk 100 m, many with mobility impairments, found the Flex to have poor ICC agreement (− 0.03 to 0.25) and underestimated steps by high levels [28]. The differences between Floegel et al.’s [28] results may be due to participants having higher mobility impairments (slower speeds) than those in the present study. This is supported by another study that found accuracy improved for the Flex at higher rather than lower speeds [34]. The ChargeHR was not validated by any of the above mentioned studies.

The ChargeHR also measured between 8 and 12 steps fewer than direct observation, but appeared to be more accurate than the Flex. Few studies have tested the validity and reliability of the ChargeHR to date [29, 30, 35, 36] and none have used older people as a sample population. In a study comparing the validity of the ChargeHR of adults (mean age 35.8 years), it was found that the ChargeHR underestimated the step count [35]. This study included only six healthy participants and asked them to walk a circuit of the local neighbourhood counting their own steps (self-report), rather than using a ‘gold standard’ measure (e.g. video observation). Low correlation was found between self-report and fitness tracker step count [35]. Only two other studies validated the ChargeHR using step count as a measure with younger adults [29, 36]. Both were conducted in a laboratory. The ChargeHR overestimated steps for 2 km/hr. walking speed and underestimated at the 3.5 km/hr. [29] in one study and underestimated steps for the track based walking and jogging exercises in the other study [36]. Underestimation of steps in this study may be due to different gait patterns of older people compared to younger adults, they may have had less dramatic movements and less clearance or acceleration which may not have been picked up as accurately by the fitness tracker devices as it is likely that these devices have been calibrated for younger populations. Findings of underestimation of steps were similar to this present study, even with the differences in age of study participants. Again if researchers choose to use the ChargeHR in future studies it must be noted that step count may be underestimated.

Fitness tracker reliability in this present study was found to be excellent between 2MWT(1) and 2MWT(2) for each device. Kooiman and colleagues [18] evaluated 10 fitness tracker devices with younger adults (mean age:39 years, standard deviation:13.1), finding similar reliability results for the Flex, however the ChargeHR was not included in their study. de Man and colleagues [35] identified good (ICC = .81) inter-device reliability for the ChargeHR worn on the dominant and non-dominant wrist. Thus, these devices may be useful in tracking changes or monitoring progress of individuals (because of the high reliability).

Results from the free-living section of this present study showed strong agreement between steps (fitness trackers) and MVPA (accelerometer) and total distance and MVPA. Physical activity minutes did not appear to synchronise across to the app correctly (e.g. 10,517 steps, 7.3 km and 0 physical activity minutes) for a number of participants (16/30). In these cases the physical activity minutes seemed low compared to the number of steps calculated. For example ID28’s fitness tracker recorded 10,517 steps (distance 7.3 km) across a 24-h period but the physical activity minutes for that day were recorded as 0 min, which does not appear plausible. This may be due to technical issues or a problem with the way some of the participants walk, unfortunately it is not possible to identify the reason. Similar results occurred for the sleep variable with 15 participants’ devices not recording sleep for the full 14-days. Eight of these were wearing a Flex which requires the person to set the device to sleep mode (multiple tapping on device), one ChargeHR participant took their device off at night and two other ChargeHR wearers had difficulty charging the device and no sleep was recorded for a few days. Therefore, data for the physical activity minutes and sleep in this present study should be viewed with caution and a more in-depth analysis of the feasibility of these devices is warranted. Hargens et al. [37] investigated 31 year older adults, 7 day step data from a Fitbit Charge (no heart rate monitor included) compared to an Actigraph GT3x + accelerometer. The Charge was found to be significantly higher than the accelerometer in average steps per day for the 7 days [37], which is in contrast to the results of the present study. The MVPA minutes across the devices in Hargen et al.’s [37] study were not significantly different.

Kooiman et al.’s [18] study in a free-living environment (devices worn for 1 day) found significant differences in step count between the ActivPAL accelerometer and the Flex, with the Flex underestimating steps by a mean difference of − 150 (limits of agreement:1424 to 1124). A free-living study which included 48 participants (average age:65.6 years, SD:6.9, 58.9% had cardiac diagnosis) who wore a Flex and Actigraph accelerometer for 4 days found the devices significantly correlated in males (*r* = .96), females (*r* = .95), all participants (*r* = .95) and cardiac patients (*r* = .95) for step count but with lower correlations for MVPA (*r* = .81; *r* = .65; *r* = .74; *r* = .71 respectively) [38]. However, in contrast to Kooiman et al.’s [18] findings, these authors found an overestimation of step count and minutes for the Flex in all participants, similar to Chu and colleagues [31] who also found an overestimation of steps by the Flex compared to the Actigraph accelerometer. Again though the participants were young adults (median age:31 years; IQR:26–42.8). The number of days fitness trackers are worn differs between many studies and this may affect the results. No studies were found that investigated reliability and validity of physical activity (step count or MVPA) of ChargeHR in a free-living environment with a population of older adults. More studies are needed to better understand the feasibility and acceptability of the Flex and ChargeHR with this population.

### Strengths and limitations

Despite the large number of studies investigating the validity of fitness trackers, this is the first known to utilise an older population in both laboratory and free-living (over 14-days) environments using the Flex and ChargeHR. Older people’s gait patterns often differ to those of younger people, therefore it is essential to determine whether fitness trackers measure these differently and if so to what effect.

This study may have limited generalisability to younger populations or those with high functional impairment, because participants were older with predominantly good mobility. Also formal evaluation of cognitive status of participants was not undertaken. Therefore, although all participants appeared to understand all instructions provided by the researchers in use of the fitness trackers, some participants may have had cognitive impairment. Future studies should formally assess cognitive status of all participants. Therefore, the results may not be generalisable across all older populations. Due to only one activity being examined in the laboratory (i.e. 2MWT) differing results may have been found if walking speed or gradient had been altered as found in other studies discussed earlier. The GENEactiv accelerometer did not measure steps and therefore we were unable to determine whether the fitness tracker devices were under- or over-estimating steps during the 14-day free-living section of the study. Devices that are not directly attached to the skin may also be affected by movement artefact or signal noise which might affect device functionality [31]. It was not possible to blind participants to the type of fitness tracker they wore during the free-living study as mentioned in the methods. This was not expected to affect the validity and reliability testing in this study but it may have affected participants’ thoughts about using a fitness tracker in the future, because the Flex shows minimal information on the device (e.g. dots for every 2000 steps) whereas the ChargeHR provides more data on the actual device. As discussed earlier issues with the synchronisation of data (e.g. physical activity minutes) were encountered. This may have been due to technical issues or gait patterns, but also could have been due to participants not synching the device correctly or being unsure how to use the app, despite being shown and given written instructions.

## Conclusion

The reliability and validity of the Flex and ChargeHR when used by older adults to measure step count is good. The ChargeHR compared to the Flex shows higher validity and reliability. Strong associations between step count and MVPA, and total distance and MVPA during free-living were also found. These two fitness trackers would be suitable for consumer use and for promoting physical activity, although both do under-estimate step counts when walking short distances.

## Abbreviations

2MWTs: Two 2 Minute Walk Tests
95%CI: 95% confidence interval
cm: Centre metre
FB: Fitbit fitness tracker
Gen: GENEactiv accelerometer
ICC: Intra Class Correlation
IQR: Inter-quartile range
kg: Kilogram
M: Mean
METs: Metabolic Equivalent of Tasks
MVPA: Moderate-to-Vigorous Physical Activity
PA: Physical activity
PASE: Physical Activity Scale for the Elderly
SD: Standard deviation
